# Crystal structure of 2-hydroxy-*N*-(2-hydroxyethyl)-*N*-{2-hydroxy-3-[(*E*)-*N*-hydroxyethanimidoyl]-5-methylbenzyl}ethanaminium acetate monohydrate

**DOI:** 10.1107/S2056989015002418

**Published:** 2015-02-18

**Authors:** Gary S. Nichol, Jamie M. Frost, Sergio Sanz, Euan K. Brechin

**Affiliations:** a School of Chemistry, The University of Edinburgh, Joseph Black Building, David Brewster Road, Edinburgh EH9 3FJ, Scotland

**Keywords:** crystal structure, hydrogen bonding, hydrate, organic salt, magnetism

## Abstract

The structure of the title hydrated mol­ecular salt, C_14_H_23_N_2_O_4_^+^·C_2_H_3_O_2_^−^·H_2_O, was determined as part of a wider study on the use of the mol­ecule as a polydentate ligand in the synthesis of Mn^III^ clusters with magnetic properties. The cation features intra­molecular O—H⋯N and N—H⋯O hydrogen-bond inter­actions. The crystal structure features a range of inter­molecular hydrogen-bonding inter­actions, principally O—H⋯O inter­actions between all three species in the asymmetric unit. An *R* ^2^_4_(8) graph-set hydrogen-bonding motif between the anion and water mol­ecules serves as a unit which links to the cation *via* the di­ethano­lamine group. Each O atom of the acetate anion accepts two hydrogen bonds.

## Related literature   

For background literature on Mn-containing single mol­ecule magnets, see: Inglis *et al.* (2012 ▸); Milios *et al.* (2007 ▸); Tasiopoulos & Perlepes (2008 ▸). For examples of the use of 3-{[bis­(2-hy­droxy­eth­yl)amino]­meth­yl}-2-hy­droxy-5-methyl­benzaldehyde in the synthesis of magnetic Mn cluster compounds, see: Sanz *et al.* (2014*a* ▸,*b* ▸) – mol­ecular wheels; Frost *et al.* (2014 ▸) – tetra­hedron cage. For examples of other magnentic oxime-containing clusters, see: Vlahopoulou *et al.* (2009 ▸); Stamatatos *et al.* (2007 ▸). For a review of pyrid­yl–oxime coordination chemistry, see: Milios *et al.* (2006 ▸). For the synthesis of 3-{[bis­(2-hy­droxy­eth­yl)amino]­meth­yl}-2-hy­droxy-5-methylbenzaldehyde, see: Wang *et al.* (2006 ▸).
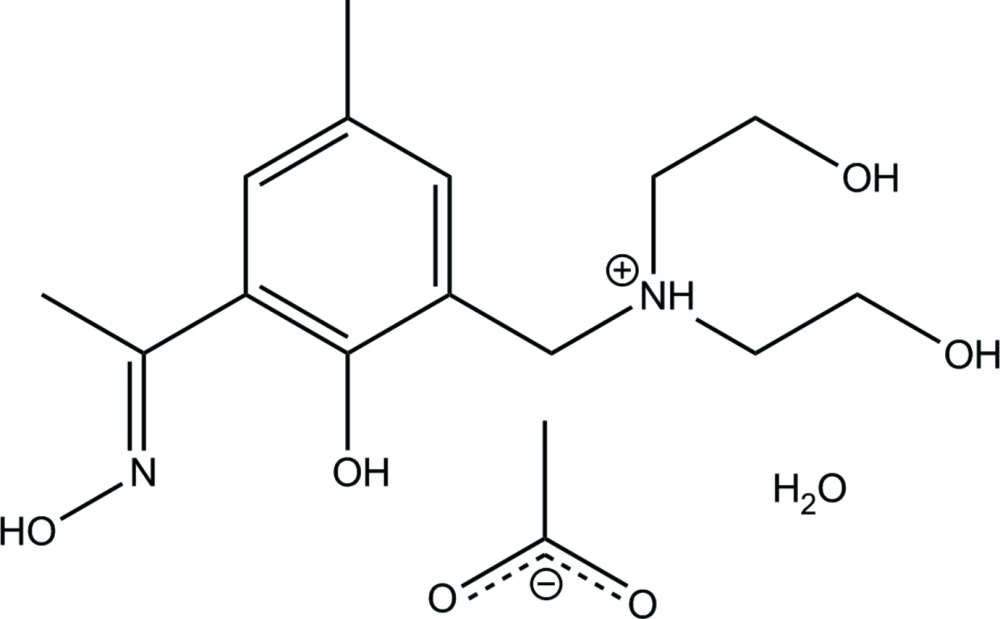


## Experimental   

### Crystal data  

C_14_H_23_N_2_O_4_^+^·C_2_H_3_O_2_^−^·H_2_O
*M* *_r_* = 360.40
Monoclinic, 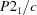

*a* = 14.4338 (5) Å
*b* = 10.4786 (3) Å
*c* = 12.4045 (4) Å
β = 101.593 (3)°
*V* = 1837.86 (10) Å^3^
*Z* = 4
Mo *K*α radiation
μ = 0.10 mm^−1^
*T* = 120 K
0.48 × 0.38 × 0.18 mm

### Data collection  

Agilent SuperNova diffractometer
Absorption correction: gaussian (*CrysAlis PRO*; Agilent, 2014 ▸) *T*_min_ = 0.942, *T*_max_ = 0.975
38067 measured reflections
5542 independent reflections
4362 reflections with *I* > 2σ(*I*)
*R*_int_ = 0.054

### Refinement  

*R*[*F* ^2^ > 2σ(*F* ^2^)] = 0.054
*wR*(*F* ^2^) = 0.129
*S* = 1.09
5542 reflections
338 parameters
All H-atom parameters refined
Δρ_max_ = 0.33 e Å^−3^
Δρ_min_ = −0.24 e Å^−3^

### 

Data collection: *CrysAlis PRO* (Agilent, 2014 ▸); cell refinement: *CrysAlis PRO*; data reduction: *CrysAlis PRO*; program(s) used to solve structure: *SHELXS97* (Sheldrick, 2008 ▸); program(s) used to refine structure: *SHELXL2014* (Sheldrick, 2015 ▸); molecular graphics: *OLEX2* (Dolomanov *et al.*, 2009 ▸); software used to prepare material for publication: *OLEX2*.

## Supplementary Material

Crystal structure: contains datablock(s) I. DOI: 10.1107/S2056989015002418/hb7350sup1.cif

Structure factors: contains datablock(s) I. DOI: 10.1107/S2056989015002418/hb7350Isup2.hkl

Supporting information file. DOI: 10.1107/S2056989015002418/hb7350Isup3.cdx

Supporting information file. DOI: 10.1107/S2056989015002418/hb7350Isup4.cml

**Fig. 1.**
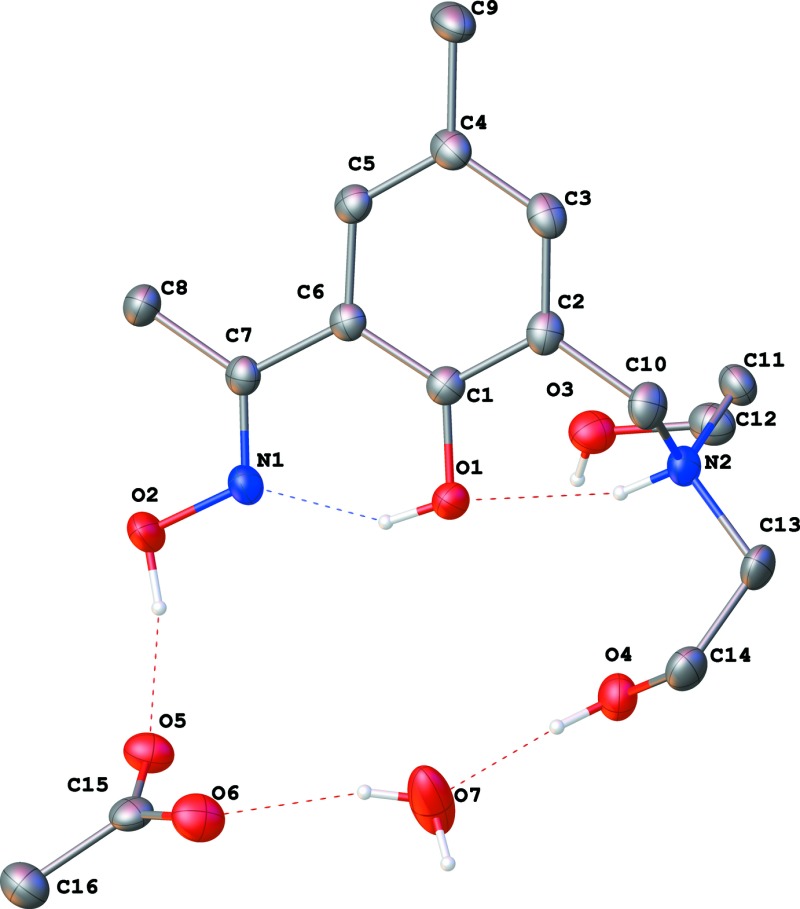
The asymmetric unit of H4L. Displacement ellipsoids are at the 50% probability level and C-bound H atoms have been omitted.

4 L . DOI: 10.1107/S2056989015002418/hb7350fig1.tif
The asymmetric unit of H_4_ *L*. Displacement ellipsoids are at the 50% probability level and C-bound H atoms have been omitted.

**Fig. 2.**
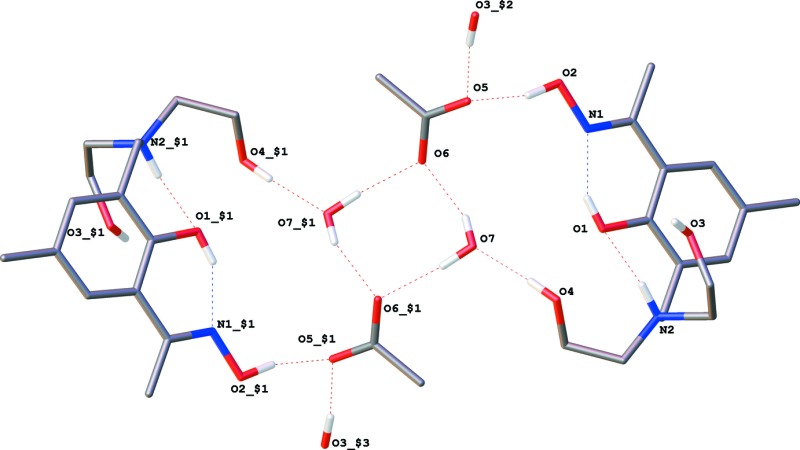
Hydrogen-bonding interactions, indicated by dashed lines, in the crystal structure of H4L. Symmetry operations for equivalent atoms: $1, 1 - x, -y, 2 - z; $2, x, 1/2 - y, 1/2 + z; $3, 1 - x, -1/2 + y, 3/2 - z.

Click here for additional data file.4 L x y z x y z x y z . DOI: 10.1107/S2056989015002418/hb7350fig2.tif
Hydrogen-bonding inter­actions, indicated by dashed lines, in the crystal structure of H_4_ *L*. Symmetry operations for equivalent atoms: $1, 1 − *x*, −*y*, 2 − *z*; $2, *x*, 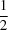 − *y*, 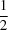 + *z*; $3, 1 − *x*, −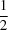 + *y*, 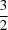 − *z*.

CCDC reference: 1047385

Additional supporting information:  crystallographic information; 3D view; checkCIF report

## Figures and Tables

**Table 1:** Hydrogen-bond geometry (, )

*D*H*A*	*D*H	H*A*	*D* *A*	*D*H*A*
O1H1N1	0.85(2)	1.78(2)	2.5368(16)	148(2)
O2H2O5	0.91(2)	1.71(2)	2.5985(16)	165(2)
O3H3O5^i^	0.82(3)	1.82(3)	2.6335(17)	171(3)
O4H4O7	0.85(2)	1.84(2)	2.6875(19)	176(2)
N2H2*A*O1	0.903(19)	2.168(18)	2.8121(16)	127.6(15)
O7H7*A*O6	0.79(3)	2.07(3)	2.823(2)	159(3)
O7H7*B*O6^ii^	0.89(3)	1.86(3)	2.738(2)	169(2)

Symmetry codes: (i) 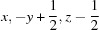; (ii) 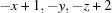.

## supplementary crystallographic information

### S1. Chemical context

The structure of the title hydrated salt was determined as part of a wider study on the synthesis of polymetallic compounds with potentially inter­esting magnetic properties. The phenolic oximes are a ligand family which have had enormous success in the construction of Mn cluster compounds that behave as single molecule magnets (Milios *et al.*, 2007; Inglis *et al.*, 2012). These ligand types tend to form systems based on the [Mn_3_O(L)_3_]^+^ (L = salicylaldoxime) building block (Vlahopoulou *et al.*, 2009; Stamatatos *et al.*, 2007; Milios *et al.*, 2006). An additional functional group was introduced onto the aromatic framework of the ligand in an attempt to disrupt the formation of clusters based on this motif and to see if higher nuclearity compounds based on phenolic oximes could be isolated. A di­ethano­lamine functional group was the obvious choice given that the this has an excellent track record of making magnetically inter­esting Mn clusters in its own right (Tasiopoulos & Perlepes, 2008). For examples of the use of the H_4_*L* in the synthesis of magnetic materials, see Frost *et al.* (2014) and Sanz *et al.* (2014a , 2014b).

### S2. Structural commentary

A check of the molecular geometry with Mogul showed all geometric parameters to be unexceptional. A mean plane fitted through atoms O1, O2, N1 and C1 to C10 (i.e. all ring atoms plus the oxime and hydroxyl groups) has an rms deviation of 0.029 Å.

### S3. Supra­molecular features

The crystal structure features extensive hydrogen bonding, principally O–H···O inter­actions involving all species in the asymmetric unit. Intra­molecular inter­actions within the cation are, perhaps, less important but serve to support the overall structure by locking the cation conformation. The N2–H2A···O1 inter­action in particular is probably quite weak. The *R*^2^_4_(8) graph set motif between the anion and water molecules serves as an important unit which links to the cation via the hy­droxy­ethane groups to propagate the three-dimensional structure. Hydrogen bonding information is summarised in Table 2.

### S4. Synthesis and crystallization

Experimental Procedures

^1^H and ^13^CNMR spectra were recorded on a nav 500 MHz spectrometer. 3-((Bis(2-hy­droxy­ethyl)­amino)­methyl)-2-hy­droxy-5-methyl­benzaldehyde was prepared according to a published procedure (Wang *et al.*, 2006). Solvents and reagents were used as received from commercial suppliers.

Synthesis of 3-{[Bis(2-hy­droxy­ethyl)­amino]­methyl}-2-hy­droxy-5-methyl­salicylaldoxime (H_4_L)

3-{[Bis(2-hy­droxy­ethyl)­amino]­methyl}-2-hy­droxy-5-methyl­benzaldehyde (10.8 g, 40 mmol), hydroxyl­amine hydro­chloride (3.5 g, 50 mmol) and sodium acetate (4.14 g, 50 mmol) were dissolved in 500 mL of ethanol. The mixture was refluxed under N_2_ for 4 h. A white precipitate was filtered off from the warm ethanol solution. The solvent was evaporated to dryness, a minimum amount of CH_2_Cl_2_ added and the sample stored at -10°C for 24 hours. Clear block shaped crystals grew and were collected after filtration (10.16 g, 90%). ^1^H NMR (500 MHz, DMSO): δ 7.12 (bs, 1H), 7.05 (bs, 1H), 3.60 (s, 2H), 3.54 (t, *J*=6.2 Hz, 4H), 2.53 (t, *J*= 6.2 Hz, 4H), 2.23 (s, 3H), 2.22 (s, 3H).^13^C NMR (500 MHz, DMSO): δ 157.28 (1C, *C*_ar_OH), 153.86 (1C, *C*NOH), 131.34 (1C, *C*H), 127.61 (1C, *C*H), 126.99 (1C, *C*), 124.34 (1C, *C*), 121.01 (1C, *C*), 59.14 (2C, *C*H_2_), 56.51 (2C, *C*H_2_), 54.78 (1C, *C*H_2_), 21.69 (1C, *C*H_3_), 12.73 (1C, *C*H_3_).

### S5. Refinement

Crystal data, data collection and structure refinement details are summarised in Table 1. All H atoms were located in a difference Fourier map and refined freely.

### Crystal data

**Table:**

C_14_H_23_N_2_O_4_^+^·C_2_H_3_O_2_^−^·H_2_O	*F*(000) = 776
*M**_r_* = 360.40	*D*_x_ = 1.303 Mg m^−^^3^
Monoclinic, *P*2_1_/*c*	Mo *K*α radiation, λ = 0.71073 Å
*a* = 14.4338 (5) Å	Cell parameters from 10187 reflections
*b* = 10.4786 (3) Å	θ = 3.5–30.2°
*c* = 12.4045 (4) Å	µ = 0.10 mm^−^^1^
β = 101.593 (3)°	*T* = 120 K
*V* = 1837.86 (10) Å^3^	Block, colourless
*Z* = 4	0.48 × 0.38 × 0.18 mm

### Data collection

**Table:**

Agilent SuperNova diffractometer	5542 independent reflections
Radiation source: SuperNova (Mo) X-ray Source	4362 reflections with *I* > 2σ(*I*)
Mirror monochromator	*R*_int_ = 0.054
Detector resolution: 5.1574 pixels mm^-1^	θ_max_ = 31.1°, θ_min_ = 3.1°
ω scans	*h* = −20→20
Absorption correction: gaussian (*CrysAlis PRO*; Agilent, 2014)	*k* = −15→13
*T*_min_ = 0.942, *T*_max_ = 0.975	*l* = −18→17
38067 measured reflections	

### Refinement

**Table:**

Refinement on *F*^2^	Primary atom site location: structure-invariant direct methods
Least-squares matrix: full	Hydrogen site location: difference Fourier map
*R*[*F*^2^ > 2σ(*F*^2^)] = 0.054	All H-atom parameters refined
*wR*(*F*^2^) = 0.129	*w* = 1/[σ^2^(*F*_o_^2^) + (0.0431*P*)^2^ + 0.9291*P*] where *P* = (*F*_o_^2^ + 2*F*_c_^2^)/3
*S* = 1.09	(Δ/σ)_max_ < 0.001
5542 reflections	Δρ_max_ = 0.33 e Å^−^^3^
338 parameters	Δρ_min_ = −0.24 e Å^−^^3^
0 restraints	

### Special details

**Table:**

Geometry. All e.s.d.'s (except the e.s.d. in the dihedral angle between two l.s. planes) are estimated using the full covariance matrix. The cell e.s.d.'s are taken into account individually in the estimation of e.s.d.'s in distances, angles and torsion angles; correlations between e.s.d.'s in cell parameters are only used when they are defined by crystal symmetry. An approximate (isotropic) treatment of cell e.s.d.'s is used for estimating e.s.d.'s involving l.s. planes.
Refinement. All H atoms were located in a difference Fourier map and refined freely.

### Fractional atomic coordinates and isotropic or equivalent isotropic displacement parameters (Å^2^)

**Table:**

	*x*	*y*	*z*	*U*_iso_*/*U*_eq_	
O1	0.25286 (7)	−0.10957 (10)	0.65849 (9)	0.0229 (2)	
H1	0.2395 (15)	−0.050 (2)	0.6990 (18)	0.046 (6)*	
O2	0.14106 (9)	0.14279 (11)	0.81376 (9)	0.0320 (3)	
H2	0.2011 (17)	0.160 (2)	0.8484 (19)	0.054 (7)*	
O3	0.24215 (8)	0.03539 (12)	0.40801 (10)	0.0304 (3)	
H3	0.2659 (18)	0.107 (3)	0.418 (2)	0.060 (7)*	
O4	0.45674 (8)	−0.04092 (11)	0.61916 (10)	0.0300 (3)	
H4	0.4552 (16)	−0.026 (2)	0.686 (2)	0.055 (7)*	
N1	0.15152 (9)	0.03976 (12)	0.74689 (10)	0.0232 (3)	
N2	0.32127 (9)	−0.19342 (12)	0.47375 (10)	0.0220 (3)	
H2A	0.3117 (13)	−0.1223 (18)	0.5112 (15)	0.028 (5)*	
C1	0.16920 (10)	−0.16143 (13)	0.60648 (11)	0.0202 (3)	
C2	0.17518 (10)	−0.26194 (13)	0.53438 (12)	0.0223 (3)	
C3	0.09294 (11)	−0.32024 (14)	0.47871 (12)	0.0246 (3)	
H3A	0.0993 (13)	−0.3890 (18)	0.4276 (15)	0.029 (5)*	
C4	0.00394 (11)	−0.27997 (14)	0.49290 (12)	0.0243 (3)	
C5	−0.00027 (10)	−0.17826 (14)	0.56389 (11)	0.0213 (3)	
H5	−0.0609 (13)	−0.1492 (17)	0.5733 (14)	0.024 (4)*	
C6	0.08079 (10)	−0.11679 (13)	0.62184 (11)	0.0192 (3)	
C7	0.07274 (10)	−0.00789 (14)	0.69504 (11)	0.0203 (3)	
C8	−0.02208 (11)	0.04239 (17)	0.70539 (14)	0.0264 (3)	
H8A	−0.0567 (16)	0.073 (2)	0.638 (2)	0.053 (6)*	
H8B	−0.0153 (17)	0.104 (2)	0.757 (2)	0.058 (7)*	
H8C	−0.0601 (16)	−0.026 (2)	0.7244 (18)	0.052 (6)*	
C9	−0.08552 (13)	−0.34031 (18)	0.43012 (14)	0.0323 (4)	
H9A	−0.1036 (15)	−0.305 (2)	0.3569 (18)	0.042 (6)*	
H9B	−0.1359 (18)	−0.334 (2)	0.474 (2)	0.063 (7)*	
H9C	−0.0770 (15)	−0.429 (2)	0.4181 (18)	0.048 (6)*	
C10	0.27174 (11)	−0.30209 (14)	0.51927 (14)	0.0261 (3)	
H10A	0.2691 (13)	−0.3701 (19)	0.4675 (16)	0.032 (5)*	
H10B	0.3144 (13)	−0.3244 (17)	0.5908 (15)	0.027 (5)*	
C11	0.27852 (12)	−0.16896 (15)	0.35473 (12)	0.0259 (3)	
H11A	0.2117 (13)	−0.1940 (17)	0.3425 (14)	0.024 (4)*	
H11B	0.3108 (13)	−0.2206 (19)	0.3119 (15)	0.032 (5)*	
C12	0.28636 (12)	−0.02870 (16)	0.33149 (13)	0.0294 (3)	
H12A	0.3503 (14)	−0.0019 (18)	0.3386 (15)	0.032 (5)*	
H12B	0.2514 (13)	−0.0128 (18)	0.2536 (15)	0.030 (5)*	
C13	0.42648 (11)	−0.21255 (17)	0.49313 (14)	0.0288 (3)	
H13A	0.4494 (12)	−0.1530 (17)	0.4413 (14)	0.025 (4)*	
H13B	0.4385 (12)	−0.3021 (18)	0.4792 (14)	0.027 (4)*	
C14	0.47242 (12)	−0.17376 (17)	0.60884 (14)	0.0298 (3)	
H14A	0.5399 (14)	−0.1927 (18)	0.6200 (15)	0.034 (5)*	
H14B	0.4445 (13)	−0.2225 (18)	0.6635 (15)	0.030 (5)*	
O5	0.30047 (8)	0.22604 (11)	0.92796 (9)	0.0297 (3)	
O6	0.38120 (9)	0.06607 (11)	1.01687 (10)	0.0349 (3)	
C15	0.34399 (10)	0.17389 (14)	1.01535 (13)	0.0242 (3)	
C16	0.35169 (15)	0.24691 (19)	1.12192 (15)	0.0347 (4)	
H16A	0.409 (2)	0.234 (3)	1.167 (3)	0.098 (11)*	
H16B	0.348 (2)	0.337 (3)	1.110 (3)	0.101 (11)*	
H16C	0.307 (2)	0.221 (3)	1.162 (3)	0.108 (12)*	
O7	0.46060 (14)	0.00383 (17)	0.83335 (12)	0.0569 (5)	
H7A	0.4274 (18)	0.028 (3)	0.873 (2)	0.059 (8)*	
H7B	0.5160 (19)	−0.016 (3)	0.875 (2)	0.064 (8)*	

### Atomic displacement parameters (Å^2^)

**Table:**

	*U*^11^	*U*^22^	*U*^33^	*U*^12^	*U*^13^	*U*^23^
O1	0.0198 (5)	0.0238 (5)	0.0247 (5)	0.0018 (4)	0.0037 (4)	−0.0027 (4)
O2	0.0276 (6)	0.0354 (6)	0.0320 (6)	0.0025 (5)	0.0035 (5)	−0.0167 (5)
O3	0.0300 (6)	0.0243 (6)	0.0361 (6)	−0.0018 (5)	0.0048 (5)	−0.0022 (5)
O4	0.0306 (6)	0.0328 (6)	0.0282 (6)	0.0025 (5)	0.0099 (5)	−0.0019 (5)
N1	0.0251 (6)	0.0229 (6)	0.0216 (6)	0.0016 (5)	0.0051 (5)	−0.0044 (5)
N2	0.0225 (6)	0.0208 (6)	0.0233 (6)	0.0035 (5)	0.0062 (5)	−0.0030 (5)
C1	0.0223 (7)	0.0177 (6)	0.0208 (6)	0.0002 (5)	0.0046 (5)	0.0031 (5)
C2	0.0268 (7)	0.0167 (6)	0.0251 (7)	0.0020 (5)	0.0096 (6)	0.0036 (5)
C3	0.0343 (8)	0.0166 (6)	0.0247 (7)	−0.0023 (6)	0.0098 (6)	0.0001 (5)
C4	0.0291 (8)	0.0232 (7)	0.0218 (6)	−0.0063 (6)	0.0077 (6)	0.0005 (5)
C5	0.0214 (7)	0.0226 (7)	0.0208 (6)	−0.0016 (5)	0.0070 (5)	0.0025 (5)
C6	0.0223 (7)	0.0180 (6)	0.0178 (6)	−0.0002 (5)	0.0053 (5)	0.0029 (5)
C7	0.0223 (7)	0.0218 (7)	0.0177 (6)	0.0008 (5)	0.0061 (5)	0.0017 (5)
C8	0.0227 (8)	0.0304 (8)	0.0271 (7)	0.0018 (6)	0.0075 (6)	−0.0024 (6)
C9	0.0333 (9)	0.0343 (9)	0.0303 (8)	−0.0124 (7)	0.0085 (7)	−0.0093 (7)
C10	0.0301 (8)	0.0185 (7)	0.0312 (8)	0.0048 (6)	0.0097 (6)	0.0005 (6)
C11	0.0294 (8)	0.0282 (8)	0.0204 (6)	−0.0018 (6)	0.0057 (6)	−0.0049 (6)
C12	0.0313 (9)	0.0310 (8)	0.0251 (7)	−0.0038 (6)	0.0039 (6)	0.0016 (6)
C13	0.0217 (8)	0.0337 (9)	0.0326 (8)	0.0081 (6)	0.0091 (6)	−0.0046 (7)
C14	0.0224 (8)	0.0337 (9)	0.0331 (8)	0.0074 (6)	0.0050 (6)	−0.0006 (7)
O5	0.0302 (6)	0.0252 (6)	0.0310 (6)	−0.0027 (4)	−0.0003 (5)	0.0003 (4)
O6	0.0394 (7)	0.0216 (6)	0.0417 (7)	0.0006 (5)	0.0032 (5)	−0.0002 (5)
C15	0.0192 (7)	0.0219 (7)	0.0311 (7)	−0.0064 (5)	0.0043 (6)	−0.0005 (6)
C16	0.0391 (10)	0.0332 (9)	0.0309 (8)	−0.0034 (7)	0.0044 (7)	−0.0042 (7)
O7	0.0679 (11)	0.0706 (11)	0.0285 (7)	0.0369 (9)	0.0011 (7)	−0.0062 (7)

### Geometric parameters (Å, º)

**Table:**

O1—H1	0.85 (2)	C8—H8B	0.91 (3)
O1—C1	1.3625 (17)	C8—H8C	0.96 (2)
O2—H2	0.91 (2)	C9—H9A	0.97 (2)
O2—N1	1.3880 (16)	C9—H9B	0.99 (3)
O3—H3	0.82 (3)	C9—H9C	0.96 (2)
O3—C12	1.415 (2)	C10—H10A	0.95 (2)
O4—H4	0.85 (2)	C10—H10B	1.001 (18)
O4—C14	1.420 (2)	C11—H11A	0.982 (18)
N1—C7	1.2891 (19)	C11—H11B	0.94 (2)
N2—H2A	0.903 (19)	C11—C12	1.506 (2)
N2—C10	1.513 (2)	C12—H12A	0.952 (19)
N2—C11	1.5034 (19)	C12—H12B	1.009 (18)
N2—C13	1.503 (2)	C13—H13A	0.999 (18)
C1—C2	1.396 (2)	C13—H13B	0.976 (19)
C1—C6	1.408 (2)	C13—C14	1.511 (2)
C2—C3	1.389 (2)	C14—H14A	0.98 (2)
C2—C10	1.503 (2)	C14—H14B	0.996 (19)
C3—H3A	0.976 (19)	O5—C15	1.2628 (18)
C3—C4	1.396 (2)	O6—C15	1.2495 (19)
C4—C5	1.392 (2)	C15—C16	1.512 (2)
C4—C9	1.506 (2)	C16—H16A	0.91 (3)
C5—H5	0.955 (18)	C16—H16B	0.96 (4)
C5—C6	1.401 (2)	C16—H16C	0.92 (4)
C6—C7	1.4773 (19)	O7—H7A	0.79 (3)
C7—C8	1.496 (2)	O7—H7B	0.89 (3)
C8—H8A	0.95 (2)		
			
C1—O1—H1	106.8 (15)	H9A—C9—H9C	104.1 (18)
N1—O2—H2	103.4 (15)	H9B—C9—H9C	106 (2)
C12—O3—H3	107.3 (18)	N2—C10—H10A	105.6 (11)
C14—O4—H4	107.4 (17)	N2—C10—H10B	104.7 (10)
C7—N1—O2	114.11 (12)	C2—C10—N2	110.81 (12)
C10—N2—H2A	107.2 (12)	C2—C10—H10A	112.3 (11)
C11—N2—H2A	106.8 (11)	C2—C10—H10B	112.1 (10)
C11—N2—C10	111.31 (12)	H10A—C10—H10B	110.8 (15)
C13—N2—H2A	106.4 (11)	N2—C11—H11A	107.6 (10)
C13—N2—C10	112.21 (12)	N2—C11—H11B	107.6 (11)
C13—N2—C11	112.46 (12)	N2—C11—C12	108.81 (12)
O1—C1—C2	116.19 (13)	H11A—C11—H11B	109.9 (15)
O1—C1—C6	123.00 (13)	C12—C11—H11A	109.9 (10)
C2—C1—C6	120.80 (13)	C12—C11—H11B	112.7 (12)
C1—C2—C10	118.00 (13)	O3—C12—C11	105.86 (13)
C3—C2—C1	119.59 (14)	O3—C12—H12A	111.0 (11)
C3—C2—C10	122.41 (13)	O3—C12—H12B	111.0 (10)
C2—C3—H3A	117.7 (11)	C11—C12—H12A	112.2 (12)
C2—C3—C4	121.35 (14)	C11—C12—H12B	107.6 (11)
C4—C3—H3A	120.9 (11)	H12A—C12—H12B	109.2 (15)
C3—C4—C9	121.51 (14)	N2—C13—H13A	105.6 (10)
C5—C4—C3	118.00 (14)	N2—C13—H13B	108.1 (10)
C5—C4—C9	120.44 (15)	N2—C13—C14	110.65 (12)
C4—C5—H5	118.6 (11)	H13A—C13—H13B	113.1 (15)
C4—C5—C6	122.59 (14)	C14—C13—H13A	107.7 (10)
C6—C5—H5	118.8 (11)	C14—C13—H13B	111.5 (10)
C1—C6—C7	121.69 (13)	O4—C14—C13	107.57 (13)
C5—C6—C1	117.66 (13)	O4—C14—H14A	111.1 (12)
C5—C6—C7	120.65 (13)	O4—C14—H14B	110.4 (11)
N1—C7—C6	115.82 (13)	C13—C14—H14A	108.3 (11)
N1—C7—C8	123.45 (13)	C13—C14—H14B	110.4 (11)
C6—C7—C8	120.73 (13)	H14A—C14—H14B	109.1 (15)
C7—C8—H8A	112.0 (14)	O5—C15—C16	117.90 (14)
C7—C8—H8B	110.1 (15)	O6—C15—O5	122.85 (15)
C7—C8—H8C	109.7 (14)	O6—C15—C16	119.25 (15)
H8A—C8—H8B	110 (2)	C15—C16—H16A	111 (2)
H8A—C8—H8C	104.4 (19)	C15—C16—H16B	112 (2)
H8B—C8—H8C	110 (2)	C15—C16—H16C	113 (2)
C4—C9—H9A	111.6 (13)	H16A—C16—H16B	105 (3)
C4—C9—H9B	109.7 (14)	H16A—C16—H16C	106 (3)
C4—C9—H9C	111.5 (13)	H16B—C16—H16C	111 (3)
H9A—C9—H9B	113.5 (19)	H7A—O7—H7B	107 (2)
			
O1—C1—C2—C3	179.65 (12)	C3—C2—C10—N2	118.43 (15)
O1—C1—C2—C10	−0.96 (19)	C3—C4—C5—C6	−0.8 (2)
O1—C1—C6—C5	−179.83 (12)	C4—C5—C6—C1	−0.1 (2)
O1—C1—C6—C7	1.0 (2)	C4—C5—C6—C7	179.07 (13)
O2—N1—C7—C6	179.25 (11)	C5—C6—C7—N1	178.18 (13)
O2—N1—C7—C8	−0.1 (2)	C5—C6—C7—C8	−2.5 (2)
N2—C11—C12—O3	−55.59 (16)	C6—C1—C2—C3	−1.3 (2)
N2—C13—C14—O4	−63.57 (17)	C6—C1—C2—C10	178.13 (13)
C1—C2—C3—C4	0.3 (2)	C9—C4—C5—C6	−178.27 (14)
C1—C2—C10—N2	−60.94 (17)	C10—N2—C11—C12	148.01 (13)
C1—C6—C7—N1	−2.68 (19)	C10—N2—C13—C14	−81.01 (16)
C1—C6—C7—C8	176.67 (13)	C10—C2—C3—C4	−179.04 (14)
C2—C1—C6—C5	1.1 (2)	C11—N2—C10—C2	−72.47 (15)
C2—C1—C6—C7	−178.03 (12)	C11—N2—C13—C14	152.57 (14)
C2—C3—C4—C5	0.7 (2)	C13—N2—C10—C2	160.50 (12)
C2—C3—C4—C9	178.13 (14)	C13—N2—C11—C12	−85.09 (16)

### Hydrogen-bond geometry (Å, º)

**Table:**

*D*—H···*A*	*D*—H	H···*A*	*D*···*A*	*D*—H···*A*
O1—H1···N1	0.85 (2)	1.78 (2)	2.5368 (16)	148 (2)
O2—H2···O5	0.91 (2)	1.71 (2)	2.5985 (16)	165 (2)
O3—H3···O5^i^	0.82 (3)	1.82 (3)	2.6335 (17)	171 (3)
O4—H4···O7	0.85 (2)	1.84 (2)	2.6875 (19)	176 (2)
N2—H2*A*···O1	0.903 (19)	2.168 (18)	2.8121 (16)	127.6 (15)
O7—H7*A*···O6	0.79 (3)	2.07 (3)	2.823 (2)	159 (3)
O7—H7*B*···O6^ii^	0.89 (3)	1.86 (3)	2.738 (2)	169 (2)

Symmetry codes: (i) *x*, −*y*+1/2, *z*−1/2; (ii) −*x*+1, −*y*, −*z*+2.
