# Supplementary figures and images for: Crystal structure of 2-hydroxy-N-(2-hydroxyethyl)-N-{2-hydroxy-3-[(E)-N-hydroxyethanimidoyl]-5-methylbenzyl}ethanaminium acetate monohydrate
Source: Acta Crystallogr E Crystallogr Commun. 2015 Feb 18;71(Pt 3):o186–7. doi: 10.1107/S2056989015002418 (PMC4350734; doi:10.1107/S2056989015002418)

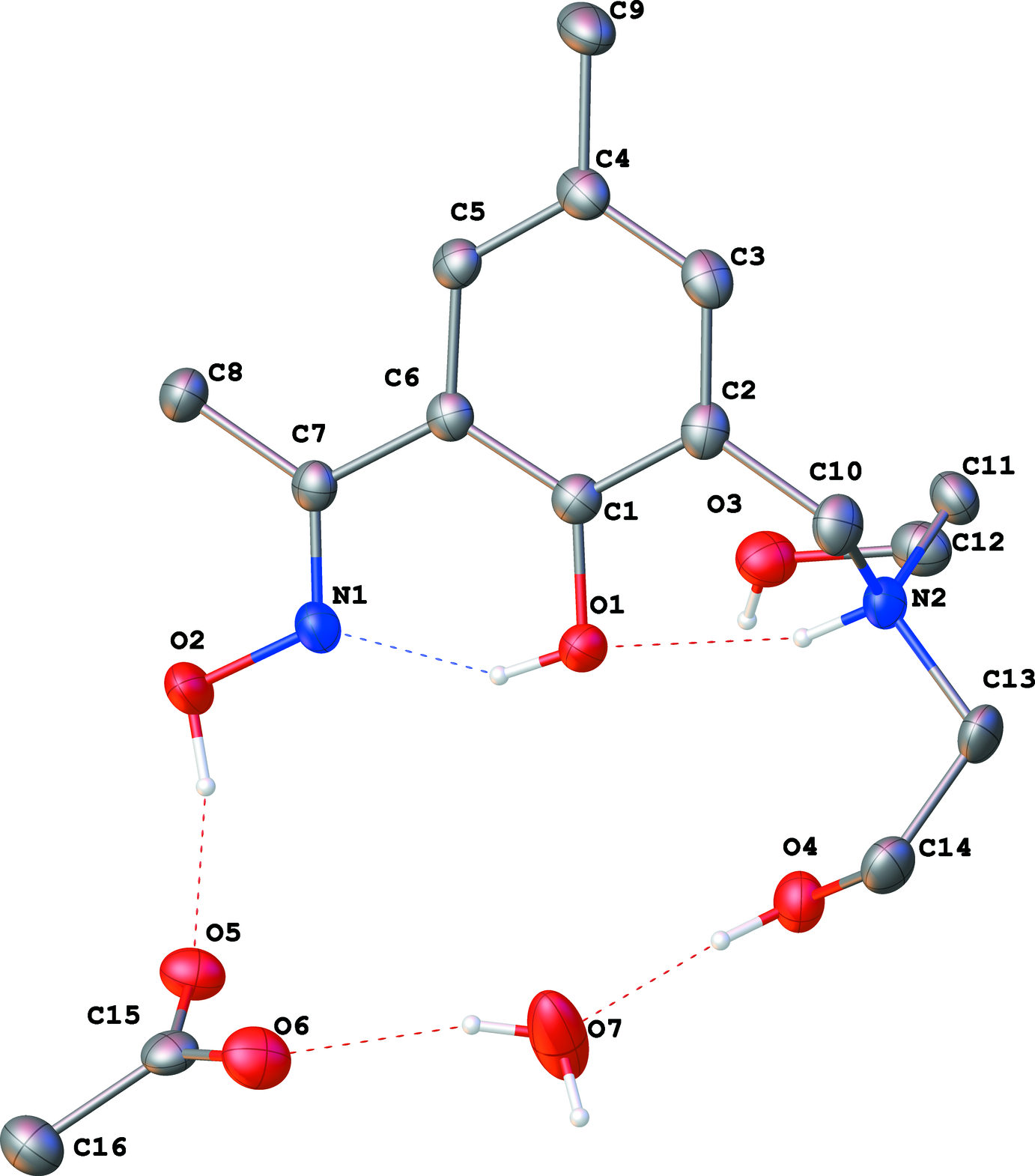

Supplement: Supplementary file 5 [file e-71-0o186-fig1.tif]

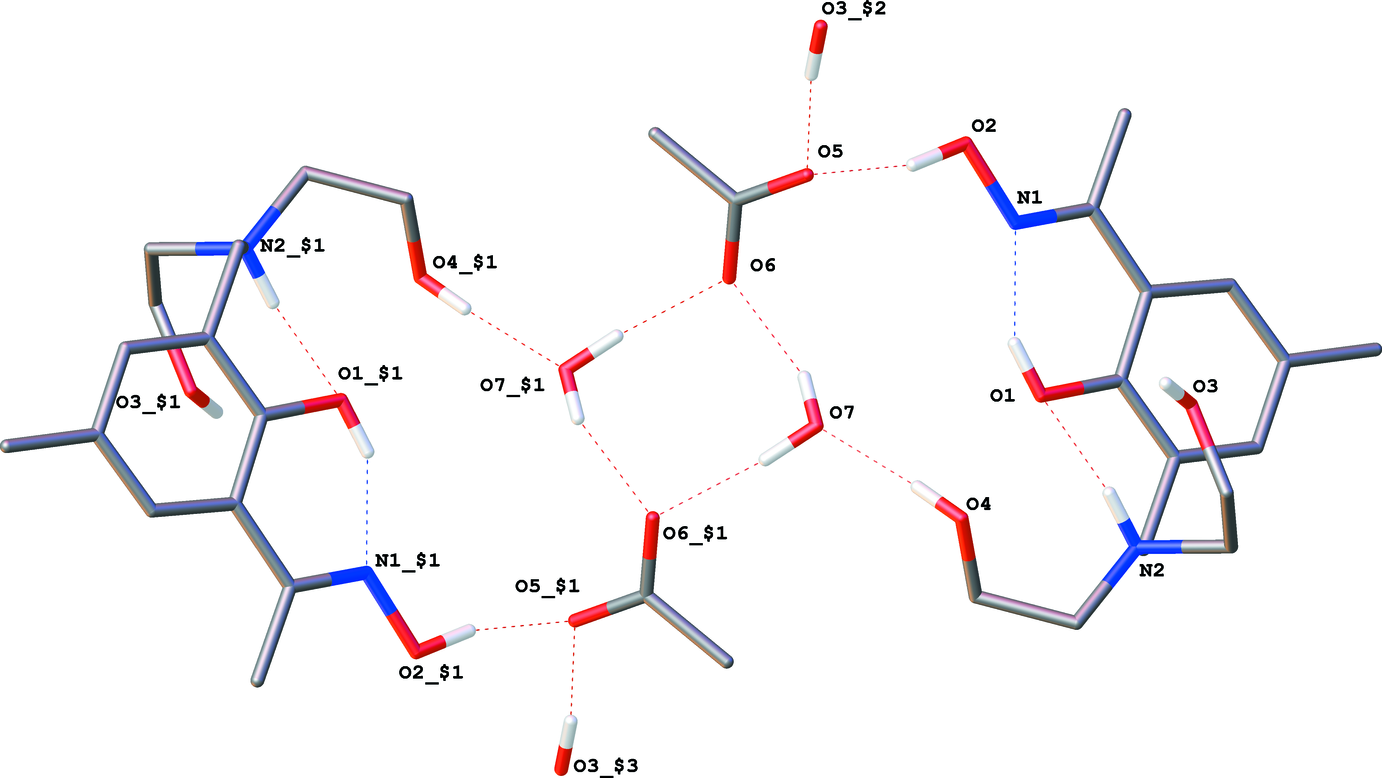

Supplement: Supplementary file 6 [file e-71-0o186-fig2.tif]
